# Flexible growing rods: polymer rods provide stability to skeletally immature spines

**DOI:** 10.1186/1748-7161-10-S1-O73

**Published:** 2015-01-19

**Authors:** Donita I Bylski-Austrow, David L Glos, Anne C Bonifas, Max F Carvalho, Matthew T Coombs, Peter F Sturm

**Affiliations:** 1 Cincinnati Children's Hospital Medical Center, USA; 2 University of Cincinnati, USA

## Objective

Surgical treatments for early onset scoliosis (EOS) typically require multiple operations and many complications. A more flexible growing rod construct might result in a more flexible spine with fewer complications. Polymer rods (polyetheretherketone, PEEK) are relatively flexible in bending, and so might allow for greater range of motion (ROM) during treatment. The purpose of this study was to determine changes in ROM of the spine after implantation of simulated growing rod constructs with a range of clinically relevant structural properties. The hypothesis was that ROM of spines instrumented with PEEK rods would be both much greater than metal rods and significantly lower than uninstrumented controls.

## Methods

Biomechanical tests were conducted on 6 skeletally immature porcine thoracic spines (domestic pigs, age 2-4 months, 35-40 kg, T1-T13). Paired pedicle screws were inserted into T3 and T4 proximally, and T10, and T11 distally. Specimens were tested under the following conditions: 1) control, then dual rods of 2) PEEK (6.25 mm, n=6), 3) titanium (4 mm, n=6), and 4) CoCr alloy (5 mm, n=4). Lateral bending (LB) and flexion-extension (FE) moments of ±5 Nm were applied. Vertebral rotations were measured using video analysis. ROM for the treated region was determined by averaging all maximum side-to-side rotations at each instrumented level. Differences were determined by two-tailed t-tests and Bonferroni post-hoc test with four primary comparisons: PEEK vs control and PEEK vs CoCr, in LB and FE (α=0.05/4).

## Results

In LB, ROM of specimens with PEEK rods was lower than control at each instrumented level. ROM was greater for PEEK rods than both Ti and CoCr at every instrumented level. Mean ROM at proximal and distal uninstrumented levels was lower for PEEK than for Ti and CoCr. In FE, mean ROM at proximal and distal uninstrumented levels was lower for PEEK than for Ti and CoCr. Combining treated levels, in LB ROM for PEEK rods was 35% of control (p<0.0001) and 270% of CoCr rods (p<0.05). In FE ROM for PEEK rods was 27% of control (p<0.005) and 180% of CoCr rods (p<0.05).

## Conclusions

PEEK rods provided increased flexibility versus metal rods, but also significantly greater stiffness than controls. Smaller increases in ROM at proximal and distal adjacent motion segments occurred with PEEK compared to the metal rods, which may decrease probability of junctional kyphosis. Flexible growing rods may form the basis of an improved treatment option for very young patients with severe spinal deformity.

